# Trajectories of length, weight, and bone mineral density among preterm infants during the first 12 months of corrected age in China

**DOI:** 10.1186/s12887-015-0396-6

**Published:** 2015-08-05

**Authors:** Zhiwei Zhao, Ming Ding, Zubin Hu, Qiong Dai, Ambika Satija, Aiqin Zhou, Yusong Xu, Xuan Zhang, Frank B. Hu, Haiqing Xu

**Affiliations:** Department of Child Health Care, Hubei Maternal and Child Health Hospital, Wuhan, Hubei 430070 China; Department of Nutrition, Harvard School of Public Health, 655 Huntington Ave, Boston, MA 02115 USA; Department of Epidemiology, Harvard School of Public Health, Boston, MA USA; Channing Division of Network Medicine, Brigham and Women’s Hospital and Harvard Medical School, Boston, MA USA

## Abstract

**Background:**

Limited evidence has been provided on the trajectories of length, weight, and bone mineral density (BMD) among preterm infants in early life in Asian countries.

**Methods:**

We conducted a longitudinal study, which included 652 late preterm (gestational age: 34–36.9 weeks), 486 moderate preterm (32–33.9), 291 very preterm (28–31.9), 149 extremely preterm infants (≤28.9) and 1434 full-term peers (≥37) during the first 12 months of corrected age in Wuhan, China. Weight and length were measured at birth, once randomly before term, and every month thereafter. BMD was examined at 3, 6, 9 and 12 months using dual-energy X-ray absorptiometry.

**Results:**

From birth to 12 months of corrected age, growth peaks in length and weight were observed at 1–3 months among preterm infants. No catch-up growth in length, weight, and BMD was observed among preterm infants. However, accelerated growth in length, weight, and BMD was found. Among extremely preterm infants, relative to full-term infants, length was −6.77 cm (95 % CI: −7.14, −6.40; P for trend < 0.001) lower during the first 12 months; weight was −1.23 kg (−1.33, −1.13; P for trend < 0.001) lower; and BMD was −0.070 g/cm^2^(−0.087, −0.053; P for trend < 0.001) lower; however, average growth rates of these measures were higher (Ps < 0.05). Small gestational age and low birth weight were independently associated with lower length, weight, and BMD.

**Conclusion:**

Growth peaks in length and weight among preterm infants were observed at 1–3 months. No catch-up growth in length, weight, and BMD was observed, however, there was accelerated growth in length, weight, and BMD.

**Electronic supplementary material:**

The online version of this article (doi:10.1186/s12887-015-0396-6) contains supplementary material, which is available to authorized users.

## Background

Due to dramatic advances in neonatal medicine, preterm infants, including extremely preterm infants (gestational age ≤ 28 weeks), are able to survive the first few weeks of life. However, preterm infants have a significantly elevated risk of death due to infection, respiratory disease, and necrotizing enterocolitis during the postnatal period [1, 2]; a higher risk of neurodevelopmental retardation [3] and autism [4], and elevated plasma insulin levels in early childhood [5]; and a higher risk of cardiovascular disease in adulthood [3]. Thus, an understanding of the early growth patterns of preterm infants may help develop appropriate daily care practices that reduce the risk of complications related to preterm birth.

A growth chart has been used to monitor infants’ length and weight at different ages [6]. For preterm infants, a growth chart from gestational age 22 to 50 weeks has been developed in Western population [7]. However, the chart uses data from cross-sectional studies, and hence cannot provide longitudinal growth trajectories of infants with specific gestational ages. Furthermore, studies have shown that, compared to full term peers, preterm infants have lower length and weight persisting into childhood [8−10]. However, whether preterm infants have accelerated growth of length and weight in early life remains to be investigated. Information on early growth rates of length and weight among preterm infants is thus needed to identify critical growth periods in this population.

In addition to length and weight, infant growth research has also focused on bone health and calcium metabolism, by measuring bone mineral density (BMD) [11]. BMD is usually assessed using dual energy x-ray absorptiometry (DEXA), which is widely accepted as a precise and accurate noninvasive method to assess the body composition of small subjects [12].

We therefore carried out a longitudinal investigation of length, weight, and BMD trajectories of late preterm, moderate preterm, very preterm, and extremely preterm infants from birth to 12 months of corrected age, and compared them with the respective trajectories of full-term peers in Wuhan, China. Our study will establish reference values of length, weight, and BMD for preterm and full-term infants in China.

## Methods

### Study population

Our participants were infants who took physical examinations within the first 12 months of corrected age at the Child Health Care Clinic of Hubei Maternal and Child Health Hospital. We excluded mothers with preeclampsia, gestational diabetes, and who reported smoking during pregnancy and infants with congenital disease, metabolic bone disease, and diagnosed chronic renal, hepatic, or gastrointestinal diseases. We further excluded participants with missing outcomes at any of the physical examinations. In total, 3012 participants were included in our study, including 652 late preterm (gestational age: 34–36.9 weeks), 486 moderate preterm (32–33.9), 291 very preterm (28–31.9), and 149 extremely preterm infants (≤28.9) and 1434 full-term peers (≥37).

Gestational age was based on the mother’s last menstrual period and first trimester ultrasonogram. For each preterm infant, age was corrected for prematurity by subtracting the number of weeks premature from the postnatal chronological age, and the number of weeks premature was calculated as 40 weeks minus the real gestational age. At least one parent of the infant was informed of the study, and written consent was signed. The study was approved by the Ethics Committee of Hubei Maternal and Child Health Hospital.

### Anthropometric measurements

Infant weight and length were measured by a trained nurse at the Department of Child Health Care of Hubei Maternal and Child Health Hospital. Electronic weighing scales were used to weigh the infants, and a length board was used to measure the length of the infants. The accuracy of weight was to 0.01 kg, and the accuracy of length was to 0.1 cm. We measured length and weight at the end of each month of age (±3 days) for all infants, and further measured length and weight once before full term for preterm infants. We measured weight and length three times per assessment period for each infant, and mean weight and length were calculated and used for further analysis.

### Bone mineral density measurement

The BMD of lumbar spine (L2-L4) was measured at 3, 6, 9, and 12 months using dual-energy X-ray absorptiometry (435A102, software version 3.8, Norland A CooperSurgical Company, USA). The measurement was carried out by a trained nurse at the Department of Child Health Care of Hubei Maternal and Child Health Hospital, and the manufacturer’s operating instructions were strictly followed. We performed a scan with the infant in a supine position without movement. If the measurement was interrupted due to the infant’s movement, a repeat scan was performed. Immobilization or swaddling was used when necessary. We performed quality control every day by calculating the variation of repeated measurements of a phantom, with coefficient of variation less than 0.9 % as acceptable precision.

### Assessment of covariates

We developed an original questionnaire which was administered to infants’ parents by trained child health care professionals through in-person interviews. Information was collected on mothers’ education, maternal age at birth, calcium supplementation during pregnancy, outdoor activity during pregnancy, gestational weight gain, gender of the infants, birth weight, and birth length.

### Data analysis

Growth rates of weight, length, and BMD were calculated as the ratio of the difference of two consecutive measurements to the time lag in months. Random-effects models were used to assess the associations of preterm birth with weight, length, and BMD and the associations of preterm birth with growth rates of weight, length, and BMD. We conducted stratified analysis by gestational week and birth weight. We further conducted stratified analysis by categorizing infants as adequate (AGA) or small for gestational age (SGA) if birth weight was ≥ 10th or < 10th percentile according to Fenton’s growth chart [7]. Statistical significance was considered as *P* < 0.05. We used SAS Version 9.2 (SAS Institute Inc., Cary, NC, USA) and R 3.1.0. for the analysis.

## Results

Baseline characteristics of participants are shown in Table 1. Preterm birth was associated with lower maternal weight gain and more inactive outdoor physical activity during pregnancy.

**Table 1:** Baseline characteristics of the infants according to gestational age

	**≥37 weeks**	**34–36.9 weeks**	**32–33.9 weeks**	**29–31.9 weeks**	**≤28.9 weeks**
Number of participants	1434	652	486	291	149
*Characteristics of mothers*					
Mothers’ education (college, %)	44	46	51	46	48
Maternal age at birth (years)	27.1 ± 4.0	27.0 ± 4.0	27.9 ± 3.5	27.6 ± 3.8	27.3 ± 3.4
Maternal weight gain (kg)	12.1 ± 3.6	9.8 ± 2.5	8.4 ± 2.4	6.7 ± 1.8	4.8 ± 1.3
Outdoor physical activity (hour/day)	1.7 ± 0.9	1.5 ± 0.8	1.3 ± 0.8	1.3 ± 0.8	1.3 ± 0.7
Calcium supplements during pregnancy (yes, %)	50	51	56	48	48
*Characteristics of infants*					
Birth weight (kg)	3.2 ± 0.3	2.7 ± 0.4	2.2 ± 0.1	1.7 ± 0.3	1.4 ± 0.3
Birth length (cm)	50.2 ± 0.2	48.1 ± 1.2	47.7 ± 1.4	46.5 ± 1.5	44.5 ± 1.5
Gender (female, %)	50	48	50	54	42

**Fig. 1:**
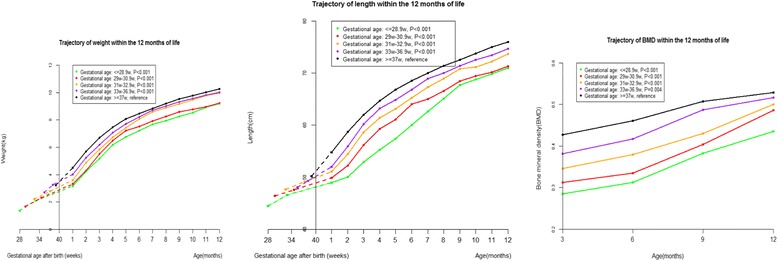
Weight, length, and bone mineral density (BMD) trajectories of preterm infants during the first 12 months of corrected age. Random-effects models adjusted for mothers’ education (less than college, college or more), calcium supplementation during pregnancy (yes, no), outdoor activity during pregnancy (hour/day, continuous), gestational weight gain (kg, continuous), maternal age at birth (year, continuous), gender of the infant (male, female), birth weight (kg, continuous), and birth length (cm, continuous)

The weight, length, and BMD of preterm infants increased with age during the first 12 months of corrected age (Fig. 1, Additional file 1: Table S1). Length, weight, and BMD were lower for preterm infants than full-term infants during the first 12 months of corrected age, with the lowest levels among extremely preterm infants (Fig. 1). For extremely preterm infants, in comparison to full term infants, length was −6.77 cm (95 % CI: −7.14, −6.40; P for trend < 0.001) lower; weight was −1.23 kg (95 % CI: −1.33, −1.13; P for trend < 0.001) lower; and BMD was −0.070 (95 % CI: (−0.087, −0.053; P for trend < 0.001) lower (Table 2).

**Table 2:** Differences in weight, length, and bone mineral density (BMD) between preterm infants and full-term peers during 1 to 12 months of corrected age

	**Gestational age**					
	**≥37 weeks**	**34–36.9 weeks**	**32–33.9 weeks**	**29–31.9 weeks**	**≤28.9 weeks**	
**Length (cm)**						*P for trend*
1–12 months	Reference	−1.60 (−1.80, −1.40)	−2.87 (−3.11, −2.63)	−4.84 (−5.11, −4.57)	−6.77 (−7.14, −6.40)	<0.001
*Stratified analysis by month*					*P for interaction*	
1–6 months	Reference	−1.98 (−2.18, −1.78)	−3.44 (−3.68, −3.20)	−5.24 (−5.53, −4.95)	−8.17 (−8.56, −7.78)	
7–12 months	Reference	−1.22 (−1.35, −1.09)	−2.30 (−2.45, −2.15)	−4.44 (−4.63, −4.25)	−5.37 (−5.62, −5.12)	<0.0001
**Weight (kg)**						*P for trend*
1–12 months	Reference	−0.29 (−0.34, −0.24)	−0.48 (−0.54, −0.42)	−1.00 (−1.08, −0.92)	−1.23 (−1.33, −1.13)	<0.001
*Stratified analysis by month*					*P for interaction*	
1–6 months	Reference	−0.39 (−0.45, −0.33)	−0.69 (−0.76, −0.62)	−1.05 (−1.14, −0.96)	−1.30 (−1.42, −1.18)	
7–12 months	Reference	−0.18 (−0.22, −0.14)	−0.27 (−0.31, −0.23)	−0.96 (−1.01, −0.91)	−1.16 (−1.23, −1.09)	<0.0001
**BMD (g/cm** ^**2**^ **)**						*P for trend*
1–12 months	Reference	−0.010 (−0.017, −0.003)	−0.040 (−0.049, −0.031)	−0.060 (−0.073, −0.047)	−0.070 (−0.087, −0.053)	<0.001
*Stratified analysis by month*					*P for interaction*	
1–6 months	Reference	−0.020 (−0.027, −0.013)	−0.050 (−0.059, −0.041)	−0.080 (−0.093, −0.067)	−0.090 (−0.11, −0.073)	
7–12 months	Reference	0.004 (−0.002, 0.011)	−0.024 (−0.033, −0.015)	−0.034 (−0.046, −0.022)	−0.054 (−0.070, −0.038)	<0.0001

Random-effects models adjusted for mothers’ education (less than college, college or more), calcium supplementation during pregnancy (yes, no), outdoor activity during pregnancy (hour/day, continuous), gestational weight gain (kg, continuous), maternal age at birth (year, continuous), gender of the infant (male, female), birth weight (kg, continuous), and birth length (cm, continuous)

**Fig. 2:**
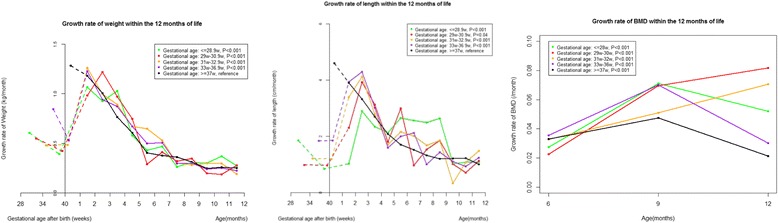
Growth rate trajectories of weight, length, and bone mineral density (BMD) of preterm infants during the first 12 months of corrected age. Random-effects models adjusted for mothers’ education (less than college, college or more), calcium supplementation during pregnancy (yes, no), outdoor activity during pregnancy (hour/day, continuous), gestational weight gain (kg, continuous), maternal age at birth (year, continuous), gender of the infant (male, female), birth weight (kg, continuous), and birth length (cm, continuous)

Differences in length, weight, and BMD between preterm birth and full term birth groups were less during 7–12 months than during 1–6 months (P for interaction: < 0.001) (Table 2). Correspondingly, length, weight, and BMD growth rates were higher among preterm infants than full term infants during 1–12 months (Fig. 2, Additional file 1: Tables S2 and S5). For preterm infants, length and weight growth rates increased after birth, peaked at 1–3 months of corrected age, and decreased thereafter. However, the growth rate of BMD did not decrease during 3 to 12 months (Fig. 2).

**Fig. 3:**
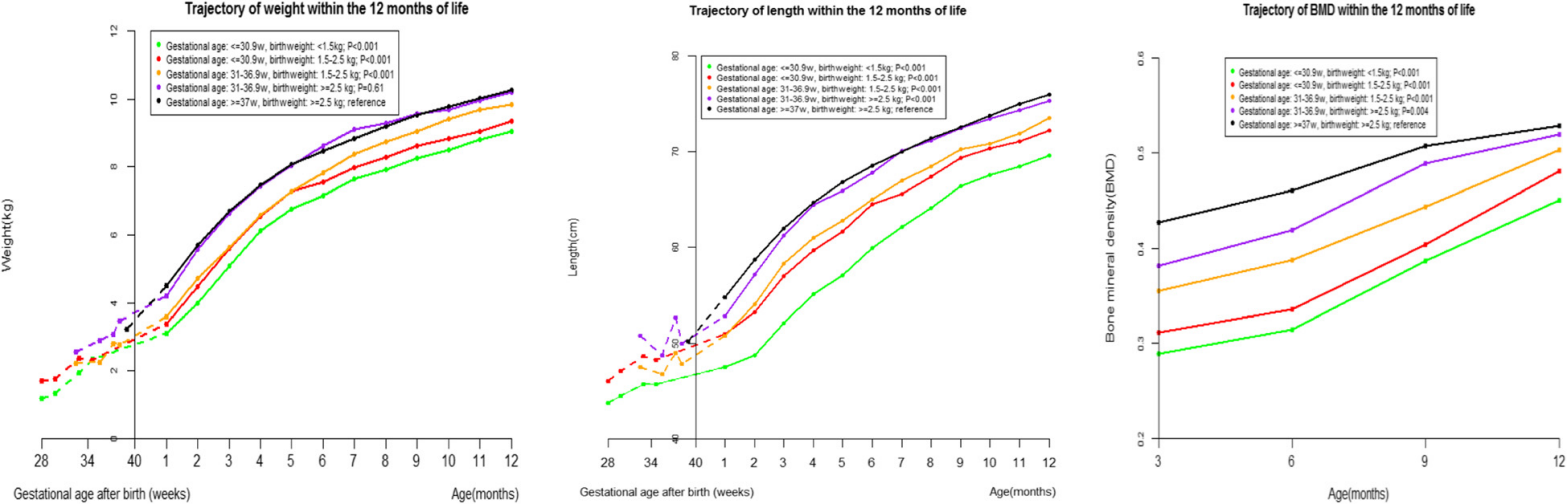
Weight, length, and bone mineral density (BMD) trajectories of infants categorized by both gestational age and birth weight during the first 12 months of corrected age. Random-effects models adjusted for mothers’ education (less than college, college or more), calcium supplementation during pregnancy (yes, no), outdoor activity during pregnancy (hour/day, continuous), gestational weight gain (kg, continuous), maternal age at birth (year, continuous), gender of the infant (male, female), birth weight (kg, continuous), and birth length (cm, continuous)

**Fig. 4:**
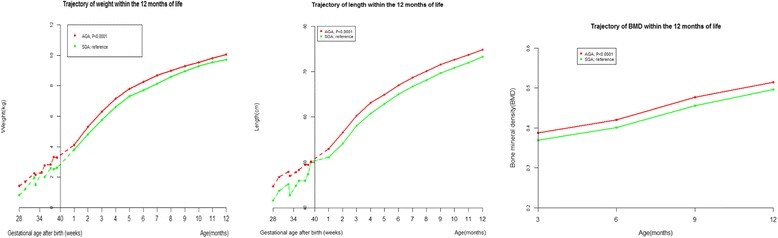
Weight, length, and bone mineral density (BMD) trajectories of adequate (AGA) or small for gestational age (SGA) preterm infants during the first 12 months of corrected age. Random-effects models adjusted for mothers’ education (less than college, college or more), calcium supplementation during pregnancy (yes, no), outdoor activity during pregnancy (hour/day, continuous), gestational weight gain (kg, continuous), maternal age at birth (year, continuous), gender of the infant (male, female), birth weight (kg, continuous), and birth length (cm, continuous)

Given only 20 of the 1434 full term infants were under 2.5 kg, we excluded those 20 infants to assess joint associations of gestational age and birth weight with length, weight, and BMD from 1 to 12 months. Smaller gestational age and lower birth weight were independently associated with lower length, weight, and BMD from 1 to 12 months (Fig. 3, Additional file 1: Tables S3 and S6). Relative to SGA, AGA was associated with higher length, weight, and BMD (Fig. 4, Additional file 1: Table S4 and S7).

## Discussion

In the present study, we described trajectories of length, weight, and BMD of 1578 preterm infants in comparison to 1434 full term peers during the first 12 months of corrected age using a prospective cohort design. We found no evidence of catch up growth in length, weight and BMD among preterm infants during the first 12 months. However, accelerated growth in length, weight, and BMD was observed, with peak growth in length and weight at 1–3 months. Smaller gestational age and lower birth weight were independently associated with lower length, weight, and BMD.

Previous studies showed that length and weight were lower for preterm infants when compared to full term peers before four years of age [8, 13]. For low birth weight preterm infants, no catch up growth in length and weight was found during the first-year [14, 15] and at 20 years of age among males [16]. Catch up growth in length was not found at 18 years of age among early preterm (<28 weeks) infants [17]. Consistently, our study showed no catch up growth during the first year among both early and late preterm infants. However, one study showed that relative to full term infants, late preterm infants had higher weight and fat mass percentage at full term and 1 month [18]. Another study showed that relative to preterm infants with higher birth weight, preterm infants with lower birth weight had lower weight and length but higher waist length ratio at full-term age [19]. Thus, the association of preterm birth, especially late preterm birth, with visceral and total adiposity needs to be further explored. We observed accelerate growth of height, weight, and BMD during follow-up period, thus, providing adequate nutrition support to satisfy the requirements of accelerated growth is important for the infant’s growth.

Our study showed growth peaks in length and weight at 1–3 months, which has important clinical implications. Evidence has shown that higher weight gain before term or before discharge was significantly associated with better neurodevelopmental and growth outcomes in later life, while extrauterine growth restriction during the first few days was recognized as a risk factor for poor neurodevelopmental outcomes [20−22]. Thus, clinical practices have been focused on nutritional support before and after the discharge of preterm infants. Randomized trials showed that when compared to a standard formula, a postdischarge formula rich in protein, calcium, phosphorus, and vitamin D could improve growth and mineralization at term, 4 months, and 6 months [23−26]. Our study indicated that nutrition support at 1–3 months of corrected age should be paid more attention as well. Whether growth rate during this critical period is associated with growth in later life warrants further investigation.

Given that there are several determinants of preterm birth and growth patterns of preterm infants might differ across these determinants, parameters such as very low birth weight and small for gestational age (SGA) have been used to discern which infants might have intrauterine growth restriction. Our study showed that SGA was associated with lower weight, length, and BMD, which is consistent with previous studies [26]. We further showed that gestational age and birth weight were independently associated with growth of the infants, indicating that both gestational age and birth weight are important parameters characterizing preterm infants.

With the popularity of DEXA as an accurate and noninvasive method, BMD has been more widely used to assess deficiency of calcium and phosphorus. Our previous study including 11,898 full term infants has provided reference values of lumbar BMD for healthy Chinese children aged 0 to 3 years [27]. Our current study not only showed that BMD of preterm infants was lower than that of full term peers, it also provided reference values during the first year for preterm infants. Interestingly, in contrast to the decreasing growth rate of length and weight from full term to 12 months, growth rate of BMD did not slow down with age. It could be that slower growth rate of length and weight is beneficial to the accumulation of minerals in the bone.

Our study has several strengths. First, we used prospective longitudinal data with a large sample size to provide valid estimates of length, weight, and BMD during the first year for infants with different gestational ages. Second, when comparing length, weight, and BMD of preterm and full-term infants, we minimized confounding by adjusting for mothers’ education, mothers’ age at birth, gestational age, weight gain during pregnancy, calcium supplement [28] and outdoor activity [27] during pregnancy, and gender of the infants. Third, to the authors’ knowledge, this is the first study to describe growth trajectories of BMD for preterm and full-term infants during the first 12 months. Lastly, we provided growth rate trajectories of length, weight, and BMD before and after term for the infants.

Our study also has several limitations. First, length and weight were measured at the end of each month of age after term, and length and weight were obtained only once randomly before term. Thus, it was difficult to obtain smooth curves of growth and growth rate and provide accurate estimates at each time point. Second, our study population was sampled from only one province in China and the growth trajectories of height, weight, and BMD of all infants might not be generalizable to other parts of China. Third, the morbidity rate of preterm infants might be higher than full term infants, however, we excluded participants who did not survive the first 12 months, resulting in potential selection bias.

## Conclusions

Our study showed no catch up growth in length, weight, and BMD among preterm infants when compared to full term peers during the first 12 months of life. However, accelerated growth in length, weight and BMD were found. Growth peaks in length and weight were observed at 1–3 months. Our study supports the provision of nutritional care during early life among preterm infants, and provides growth references for this population.

## Additional file

Additional file 1: Table S1. Weight, length, and bone mineral density (BMD) of preterm newborns within the first 12 months of corrected age. **Table S2.** Growth rate of weight, length, and bone mineral density (BMD) of preterm newborns within the first 12 months of corrected age. **Table S3.** Weight, length, and bone mineral density (BMD) of newborns categorized by both gestational age and birth weight within the first 12 months of corrected age. **Table S4.** Weight, length, and bone mineral density (BMD) of adequate (AGA) or small for gestational age (SGA) preterm infants within 1 to 12 months of corrected age. **Table S5.** Differences of growth rates of weight, length, and bone mineral density (BMD) of preterm newborns when compared with full-term peers within 1 to 12 months of corrected age. **Table S6.** Differences of growth rate of weight, length, and bone mineral density (BMD) of preterm newborns categorized by gestational age (GA) and birth weight (BW) within 1 to 12 months of corrected age. **Table S7.** Differences in growth rate of weight, length, and bone mineral density (BMD) between adequate (AGA) and small for gestational age (SGA) preterm infants within 1 to 12 months of corrected age.

## Abbreviations

BMD: Bone mineral density
SGA: Gestational age
DEXA: Dual energy x-ray absorptiometry
