# Supplementary material for: Trajectories of length, weight, and bone mineral density among preterm infants during the first 12 months of corrected age in China
Source: BMC Pediatr. 2015 Aug 5;15:91. doi: 10.1186/s12887-015-0396-6 (PMC4526172; doi:10.1186/s12887-015-0396-6)
Supplement: Additional file 1: Table S1. — Weight, length, and bone mineral density (BMD) of preterm newborns within the first 12 months of corrected age. Table S2. Growth rate of weight, length, and bone mineral density (BMD) of preterm newborns within the first 12 months of corrected age. Table S3. Weight, length, and bone mineral density (BMD) of newborns categorized by both gestational age and birth weight within the first 12 months of corrected age. Table S4. Weight, length, and bone mineral density (BMD) of adequate (AGA) or small for gestational age (SGA) preterm infants within 1 to 12 months of corrected age. Table S5. Differences of growth rates of weight, length, and bone mineral density (BMD) of preterm newborns when compared with full-term peers within 1 to 12 months of corrected age. Table S6. Differences of growth rate of weight, length, and bone mineral density (BMD) of preterm newborns categorized by gestational age (GA) and birth weight (BW) within 1 to 12 months of corrected age. Table S7. Differences in growth rate of weight, length, and bone mineral density (BMD) between adequate (AGA) and small for gestational age (SGA) preterm infants within 1 to 12 months of corrected age. [file 12887_2015_396_MOESM1_ESM.docx]

Table S1. Weight, length, and bone mineral density (BMD) of preterm newborns within the first 12 months of corrected age.

| Weight | | | | Length | | | | BMD | | | |
| --- | --- | --- | --- | --- | --- | --- | --- | --- | --- | --- | --- |
| Infant age (month) | Gestational week | Mean  (kg) | SD  (kg) | Infant age  (month) | Gestational week | Mean  (cm) | SD  (cm) | Infant age  (month) | Gestational week | Mean | SD |
| Before full term |  |  |  | Before full term |  |  |  |  |  |  |  |
| 37.0 | ≤ 28.9 | 1.4 | 0.3 | 37.0 | ≤ 28.9 | 44.5 | 1.5 |  |  |  |  |
| 37.5 | 29-31.9 | 1.7 | 0.3 | 37.5 | 29-31.9 | 46.5 | 1.5 |  |  |  |  |
| 38.1 | 32-33.9 | 2.2 | 0.1 | 38.1 | 32-33.9 | 47.7 | 1.4 |  |  |  |  |
| 38.2 | ≤ 28.9 | 2.1 | 0.4 | 38.2 | ≤ 28.9 | 46.7 | 2.0 |  |  |  |  |
| 38.7 | 29-31.9 | 2.3 | 0.2 | 38.7 | 29-31.9 | 47.6 | 1.6 |  |  |  |  |
| 38.9 | 34-36.9 | 2.7 | 0.4 | 38.9 | 34-36.9 | 48.1 | 1.2 |  |  |  |  |
| 39.3 | 32-33.9 | 2.8 | 0.1 | 39.3 | 32-33.9 | 49.1 | 1.8 |  |  |  |  |
| 39.5 | 34-36.9 | 3.2 | 0.4 | 39.5 | 34-36.9 | 49.3 | 1.3 |  |  |  |  |
| 39.8 | ≥ 37 | 3.2 | 0.3 | 39.8 | ≥ 37 | 50.2 | 0.2 |  |  |  |  |
| After full term |  |  |  | After full term |  |  |  | After full term |  |  |  |
| 1 month | ≤ 28.9 | 3.2 | 0.4 | 1 month | ≤ 28.9 | 49.0 | 2.6 | 3 month | ≤ 28.9 | 0.3 | 0.1 |
| 1 month | 29-31.9 | 3.3 | 0.2 | 1 month | 29-31.9 | 49.9 | 2.1 | 3 month | 29-31.9 | 0.3 | 0.1 |
| 1 month | 32-33.9 | 3.6 | 0.4 | 1 month | 32-33.9 | 51.1 | 2.4 | 3 month | 32-33.9 | 0.3 | 0.1 |
| 1 month | 34-36.9 | 4.0 | 0.4 | 1 month | 34-36.9 | 52.0 | 1.4 | 3 month | 34-36.9 | 0.4 | 0.1 |
| 1 month | ≥ 37 | 4.5 | 0.3 | 1 month | ≥ 37 | 54.8 | 0.8 | 3 month | ≥ 37 | 0.4 | 0.0 |
| 2 month | ≤ 28.9 | 4.2 | 0.9 | 2 month | ≤ 28.9 | 50.0 | 2.6 | 6 month | ≤ 28.9 | 0.3 | 0.1 |
| 2 month | 29-31.9 | 4.3 | 0.4 | 2 month | 29-31.9 | 52.2 | 2.6 | 6 month | 29-31.9 | 0.3 | 0.1 |
| 2 month | 32-33.9 | 4.9 | 0.2 | 2 month | 32-33.9 | 54.5 | 2.1 | 6 month | 32-33.9 | 0.4 | 0.1 |
| 2 month | 34-36.9 | 5.2 | 0.7 | 2 month | 34-36.9 | 55.9 | 2.3 | 6 month | 34-36.9 | 0.4 | 0.0 |
| 2 month | ≥ 37 | 5.7 | 0.3 | 2 month | ≥ 37 | 58.7 | 0.8 | 6 month | ≥ 37 | 0.5 | 0.0 |
| 3 month | ≤ 28.9 | 5.2 | 0.7 | 3 month | ≤ 28.9 | 52.9 | 2.9 | 9 month | ≤ 28.9 | 0.4 | 0.1 |
| 3 month | 29-31.9 | 5.5 | 0.5 | 3 month | 29-31.9 | 56.2 | 2.4 | 9 month | 29-31.9 | 0.4 | 0.1 |
| 3 month | 32-33.9 | 5.9 | 0.5 | 3 month | 32-33.9 | 58.6 | 2.2 | 9 month | 32-33.9 | 0.4 | 0.1 |
| 3 month | 34-36.9 | 6.2 | 0.9 | 3 month | 34-36.9 | 60.2 | 2.0 | 9 month | 34-36.9 | 0.5 | 0.1 |
| 3 month | ≥ 37 | 6.7 | 0.3 | 3 month | ≥ 37 | 62.0 | 0.8 | 9 month | ≥ 37 | 0.5 | 0.0 |
| 4 month | ≤ 28.9 | 6.2 | 0.5 | 4 month | ≤ 28.9 | 55.3 | 2.5 | 12 month | ≤ 28.9 | 0.4 | 0.1 |
| 4 month | 29-31.9 | 6.5 | 0.6 | 4 month | 29-31.9 | 59.3 | 2.0 | 12 month | 29-31.9 | 0.5 | 0.1 |
| 4 month | 32-33.9 | 6.7 | 0.7 | 4 month | 32-33.9 | 61.4 | 2.4 | 12 month | 32-33.9 | 0.5 | 0.1 |
| 4 month | 34-36.9 | 7.1 | 0.8 | 4 month | 34-36.9 | 63.3 | 2.3 | 12 month | 34-36.9 | 0.5 | 0.0 |
| 4 month | ≥ 37 | 7.5 | 0.3 | 4 month | ≥ 37 | 64.7 | 0.8 | 12 month | ≥ 37 | 0.5 | 0.0 |
| 5 month | ≤ 28.9 | 6.8 | 0.5 | 5 month | ≤ 28.9 | 57.4 | 2.6 |  |  |  |  |
| 5 month | 29-31.9 | 7.2 | 0.4 | 5 month | 29-31.9 | 61.1 | 1.9 |  |  |  |  |
| 5 month | 32-33.9 | 7.4 | 0.7 | 5 month | 32-33.9 | 63.1 | 2.0 |  |  |  |  |
| 5 month | 34-36.9 | 7.7 | 0.6 | 5 month | 34-36.9 | 64.8 | 2.0 |  |  |  |  |
| 5 month | ≥ 37 | 8.1 | 0.2 | 5 month | ≥ 37 | 66.8 | 0.6 |  |  |  |  |
| 6 month | ≤ 28.9 | 7.2 | 0.5 | 6 month | ≤ 28.9 | 60.1 | 2.3 |  |  |  |  |
| 6 month | 29-31.9 | 7.5 | 0.3 | 6 month | 29-31.9 | 64.1 | 2.0 |  |  |  |  |
| 6 month | 32-33.9 | 8.1 | 0.7 | 6 month | 32-33.9 | 65.3 | 2.0 |  |  |  |  |
| 6 month | 34-36.9 | 8.2 | 0.8 | 6 month | 34-36.9 | 66.8 | 1.8 |  |  |  |  |
| 6 month | ≥ 37 | 8.5 | 0.2 | 6 month | ≥ 37 | 68.5 | 0.5 |  |  |  |  |
| 7 month | ≤ 28.9 | 7.7 | 0.4 | 7 month | ≤ 28.9 | 62.6 | 1.9 |  |  |  |  |
| 7 month | 29-31.9 | 7.9 | 0.4 | 7 month | 29-31.9 | 65.1 | 1.8 |  |  |  |  |
| 7 month | 32-33.9 | 8.6 | 0.8 | 7 month | 32-33.9 | 67.3 | 2.0 |  |  |  |  |
| 7 month | 34-36.9 | 8.7 | 0.7 | 7 month | 34-36.9 | 69.0 | 2.1 |  |  |  |  |
| 7 month | ≥ 37 | 8.8 | 0.2 | 7 month | ≥ 37 | 70.0 | 0.3 |  |  |  |  |
| 8 month | ≤ 28.9 | 7.9 | 0.4 | 8 month | ≤ 28.9 | 65.1 | 1.9 |  |  |  |  |
| 8 month | 29-31.9 | 8.2 | 0.2 | 8 month | 29-31.9 | 66.6 | 1.9 |  |  |  |  |
| 8 month | 32-33.9 | 8.9 | 0.6 | 8 month | 32-33.9 | 69.0 | 1.8 |  |  |  |  |
| 8 month | 34-36.9 | 9.0 | 0.6 | 8 month | 34-36.9 | 70.0 | 2.2 |  |  |  |  |
| 8 month | ≥ 37 | 9.2 | 0.2 | 8 month | ≥ 37 | 71.4 | 0.3 |  |  |  |  |
| 9 month | ≤ 28.9 | 8.2 | 0.4 | 9 month | ≤ 28.9 | 67.8 | 2.0 |  |  |  |  |
| 9 month | 29-31.9 | 8.6 | 0.3 | 9 month | 29-31.9 | 68.4 | 1.9 |  |  |  |  |
| 9 month | 32-33.9 | 9.2 | 0.6 | 9 month | 32-33.9 | 70.8 | 1.9 |  |  |  |  |
| 9 month | 34-36.9 | 9.3 | 0.4 | 9 month | 34-36.9 | 71.4 | 1.9 |  |  |  |  |
| 9 month | ≥ 37 | 9.5 | 0.1 | 9 month | ≥ 37 | 72.6 | 0.4 |  |  |  |  |
| 10 month | ≤ 28.9 | 8.5 | 0.3 | 10 month | ≤ 28.9 | 68.8 | 1.9 |  |  |  |  |
| 10 month | 29-31.9 | 8.8 | 0.3 | 10 month | 29-31.9 | 69.5 | 1.8 |  |  |  |  |
| 10 month | 32-33.9 | 9.5 | 0.4 | 10 month | 32-33.9 | 71.2 | 1.7 |  |  |  |  |
| 10 month | 34-36.9 | 9.6 | 0.3 | 10 month | 34-36.9 | 72.5 | 1.8 |  |  |  |  |
| 10 month | ≥ 37 | 9.8 | 0.2 | 10 month | ≥ 37 | 73.8 | 0.3 |  |  |  |  |
| 11 month | ≤ 28.9 | 8.9 | 0.4 | 11 month | ≤ 28.9 | 69.9 | 1.6 |  |  |  |  |
| 11 month | 29-31.9 | 9.0 | 0.4 | 11 month | 29-31.9 | 70.2 | 1.9 |  |  |  |  |
| 11 month | 32-33.9 | 9.8 | 0.5 | 11 month | 32-33.9 | 72.2 | 1.6 |  |  |  |  |
| 11 month | 34-36.9 | 9.8 | 0.3 | 11 month | 34-36.9 | 73.4 | 1.8 |  |  |  |  |
| 11 month | ≥ 37 | 10.0 | 0.2 | 11 month | ≥ 37 | 75.0 | 0.3 |  |  |  |  |
| 12 month | ≤ 28.9 | 9.2 | 0.5 | 12 month | ≤ 28.9 | 70.9 | 1.5 |  |  |  |  |
| 12 month | 29-31.9 | 9.2 | 0.2 | 12 month | 29-31.9 | 71.3 | 1.9 |  |  |  |  |
| 12 month | 32-33.9 | 10.0 | 0.6 | 12 month | 32-33.9 | 73.7 | 1.4 |  |  |  |  |
| 12 month | 34-36.9 | 10.0 | 0.3 | 12 month | 34-36.9 | 74.7 | 1.4 |  |  |  |  |
| 12 month | ≥ 37 | 10.3 | 0.2 | 12 month | ≥ 37 | 76.0 | 0.4 |  |  |  |  |

Table S2. Growth rate of weight, length, and bone mineral density (BMD) of preterm newborns within the first 12 months of corrected age.

| Growth rate of weight | | |  | Growth rate of length | | |  | Growth rate of BMD | | |  |
| --- | --- | --- | --- | --- | --- | --- | --- | --- | --- | --- | --- |
| Gestational week | Month | Mean  (kg) | SD  (kg) | Gestational week | Month | Mean  (cm) | SD  (cm) | Gestational week | Month | Mean | SD |
| Before full term |  |  |  | Before full term |  |  |  |  |  |  |  |
| ≤ 28.9 | 39.6 | 0.4 | 0.1 | ≤ 28.9 | 39.6 | 0.9 | 0.3 |  |  |  |  |
| 29-31.9 | 39.8 | 0.4 | 0.1 | 29-31.9 | 39.8 | 1.0 | 0.6 |  |  |  |  |
| After full term |  |  |  | After full term |  |  |  | After full term |  |  |  |
| 32-33.9 | 0.2 | 0.5 | 0.3 | 32-33.9 | 0.2 | 1.2 | 0.5 | ≤ 28.9 | 6.0 | 0.03 | 0.02 |
| 34-36.9 | 0.3 | 0.5 | 0.1 | 34-36.9 | 0.3 | 1.9 | 0.3 | 29-31.9 | 6.0 | 0.02 | 0.02 |
| ≥ 37 | 0.4 | 1.3 | 0.4 | ≥ 37 | 0.4 | 4.6 | 0.6 | 32-33.9 | 6.0 | 0.03 | 0.01 |
| ≤ 28.9 | 1.5 | 1.1 | 0.5 | ≤ 28.9 | 1.5 | 1.0 | 0.4 | 34-36.9 | 6.0 | 0.04 | 0.01 |
| 29-31.9 | 1.5 | 1.0 | 0.2 | 29-31.9 | 1.5 | 2.3 | 0.6 | ≥ 37 | 6.0 | 0.03 | 0.00 |
| 32-33.9 | 1.5 | 1.3 | 0.2 | 32-33.9 | 1.5 | 3.4 | 0.4 | ≤ 28.9 | 9.0 | 0.07 | 0.03 |
| 34-36.9 | 1.5 | 1.2 | 0.3 | 34-36.9 | 1.5 | 3.9 | 1.0 | 29-31.9 | 9.0 | 0.07 | 0.04 |
| ≥ 37 | 1.5 | 1.2 | 0.0 | ≥ 37 | 1.5 | 3.9 | 1.1 | 32-33.9 | 9.0 | 0.05 | 0.01 |
| ≤ 28.9 | 2.5 | 0.9 | 0.2 | ≤ 28.9 | 2.5 | 2.9 | 0.5 | 34-36.9 | 9.0 | 0.07 | 0.01 |
| 29-31.9 | 2.5 | 1.2 | 0.2 | 29-31.9 | 2.5 | 3.9 | 0.3 | ≥ 37 | 9.0 | 0.05 | 0.01 |
| 32-33.9 | 2.5 | 1.0 | 0.3 | 32-33.9 | 2.5 | 4.1 | 0.2 | ≤ 28.9 | 12.0 | 0.05 | 0.01 |
| 34-36.9 | 2.5 | 0.9 | 0.2 | 34-36.9 | 2.5 | 4.3 | 0.4 | 29-31.9 | 12.0 | 0.08 | 0.03 |
| ≥ 37 | 2.5 | 1.0 | 0.0 | ≥ 37 | 2.5 | 3.3 | 1.1 | 32-33.9 | 12.0 | 0.07 | 0.02 |
| ≤ 28.9 | 3.5 | 1.0 | 0.2 | ≤ 28.9 | 3.5 | 2.3 | 0.5 | 34-36.9 | 12.0 | 0.03 | 0.01 |
| 29-31.9 | 3.5 | 1.0 | 0.2 | 29-31.9 | 3.5 | 3.1 | 0.5 | ≥ 37 | 12.0 | 0.02 | 0.00 |
| 32-33.9 | 3.5 | 0.9 | 0.2 | 32-33.9 | 3.5 | 2.8 | 0.3 |  |  |  |  |
| 34-36.9 | 3.5 | 0.9 | 0.2 | 34-36.9 | 3.5 | 3.0 | 0.4 |  |  |  |  |
| ≥ 37 | 3.5 | 0.8 | 0.0 | ≥ 37 | 3.5 | 2.7 | 0.9 |  |  |  |  |
| ≤ 28.9 | 4.5 | 0.6 | 0.1 | ≤ 28.9 | 4.5 | 2.1 | 0.5 |  |  |  |  |
| 29-31.9 | 4.5 | 0.7 | 0.2 | 29-31.9 | 4.5 | 1.8 | 0.2 |  |  |  |  |
| 32-33.9 | 4.5 | 0.7 | 0.1 | 32-33.9 | 4.5 | 1.7 | 0.4 |  |  |  |  |
| 34-36.9 | 4.5 | 0.7 | 0.2 | 34-36.9 | 4.5 | 1.6 | 0.3 |  |  |  |  |
| ≥ 37 | 4.5 | 0.6 | 0.1 | ≥ 37 | 4.5 | 2.1 | 0.8 |  |  |  |  |
| ≤ 28.9 | 5.5 | 0.4 | 0.1 | ≤ 28.9 | 5.5 | 2.6 | 0.5 |  |  |  |  |
| 29-31.9 | 5.5 | 0.3 | 0.1 | 29-31.9 | 5.5 | 3.0 | 0.2 |  |  |  |  |
| 32-33.9 | 5.5 | 0.6 | 0.1 | 32-33.9 | 5.5 | 2.2 | 0.2 |  |  |  |  |
| 34-36.9 | 5.5 | 0.5 | 0.2 | 34-36.9 | 5.5 | 2.0 | 0.2 |  |  |  |  |
| ≥ 37 | 5.5 | 0.4 | 0.1 | ≥ 37 | 5.5 | 1.7 | 0.6 |  |  |  |  |
| ≤ 28.9 | 6.5 | 0.5 | 0.1 | ≤ 28.9 | 6.5 | 2.6 | 0.5 |  |  |  |  |
| 29-31.9 | 6.5 | 0.4 | 0.1 | 29-31.9 | 6.5 | 1.0 | 0.3 |  |  |  |  |
| 32-33.9 | 6.5 | 0.5 | 0.1 | 32-33.9 | 6.5 | 2.0 | 0.1 |  |  |  |  |
| 34-36.9 | 6.5 | 0.5 | 0.1 | 34-36.9 | 6.5 | 2.1 | 0.3 |  |  |  |  |
| ≥ 37 | 6.5 | 0.4 | 0.0 | ≥ 37 | 6.5 | 1.5 | 0.5 |  |  |  |  |
| ≤ 28.9 | 7.5 | 0.3 | 0.0 | ≤ 28.9 | 7.5 | 2.5 | 0.2 |  |  |  |  |
| 29-31.9 | 7.5 | 0.3 | 0.2 | 29-31.9 | 7.5 | 1.5 | 0.2 |  |  |  |  |
| 32-33.9 | 7.5 | 0.3 | 0.1 | 32-33.9 | 7.5 | 1.7 | 0.2 |  |  |  |  |
| 34-36.9 | 7.5 | 0.3 | 0.2 | 34-36.9 | 7.5 | 1.0 | 0.1 |  |  |  |  |
| ≥ 37 | 7.5 | 0.4 | 0.0 | ≥ 37 | 7.5 | 1.3 | 0.4 |  |  |  |  |
| ≤ 28.9 | 8.5 | 0.3 | 0.1 | ≤ 28.9 | 8.5 | 2.6 | 0.3 |  |  |  |  |
| 29-31.9 | 8.5 | 0.3 | 0.1 | 29-31.9 | 8.5 | 1.9 | 0.1 |  |  |  |  |
| 32-33.9 | 8.5 | 0.3 | 0.1 | 32-33.9 | 8.5 | 1.9 | 0.2 |  |  |  |  |
| 34-36.9 | 8.5 | 0.3 | 0.1 | 34-36.9 | 8.5 | 1.4 | 0.3 |  |  |  |  |
| ≥ 37 | 8.5 | 0.3 | 0.1 | ≥ 37 | 8.5 | 1.2 | 0.4 |  |  |  |  |
| ≤ 28.9 | 9.5 | 0.3 | 0.1 | ≤ 28.9 | 9.5 | 1.0 | 0.3 |  |  |  |  |
| 29-31.9 | 9.5 | 0.2 | 0.0 | 29-31.9 | 9.5 | 1.0 | 0.2 |  |  |  |  |
| 32-33.9 | 9.5 | 0.3 | 0.1 | 32-33.9 | 9.5 | 0.3 | 0.3 |  |  |  |  |
| 34-36.9 | 9.5 | 0.2 | 0.2 | 34-36.9 | 9.5 | 1.1 | 0.2 |  |  |  |  |
| ≥ 37 | 9.5 | 0.2 | 0.1 | ≥ 37 | 9.5 | 1.2 | 0.4 |  |  |  |  |
| ≤ 28.9 | 10.5 | 0.4 | 0.1 | ≤ 28.9 | 10.5 | 1.1 | 0.3 |  |  |  |  |
| 29-31.9 | 10.5 | 0.2 | 0.1 | 29-31.9 | 10.5 | 0.7 | 0.2 |  |  |  |  |
| 32-33.9 | 10.5 | 0.3 | 0.1 | 32-33.9 | 10.5 | 1.1 | 0.2 |  |  |  |  |
| 34-36.9 | 10.5 | 0.3 | 0.0 | 34-36.9 | 10.5 | 0.9 | 0.1 |  |  |  |  |
| ≥ 37 | 10.5 | 0.3 | 0.0 | ≥ 37 | 10.5 | 1.2 | 0.4 |  |  |  |  |
| ≤ 28.9 | 11.5 | 0.3 | 0.1 | ≤ 28.9 | 11.5 | 1.1 | 0.2 |  |  |  |  |
| 29-31.9 | 11.5 | 0.3 | 0.2 | 29-31.9 | 11.5 | 1.1 | 0.1 |  |  |  |  |
| 32-33.9 | 11.5 | 0.2 | 0.1 | 32-33.9 | 11.5 | 1.5 | 0.3 |  |  |  |  |
| 34-36.9 | 11.5 | 0.2 | 0.1 | 34-36.9 | 11.5 | 1.2 | 0.4 |  |  |  |  |
| ≥ 37 | 11.5 | 0.3 | 0.0 | ≥ 37 | 11.5 | 1.0 | 0.2 |  |  |  |  |

Table S3. Weight, length, and bone mineral density (BMD) of newborns categorized by both gestational age and birth weight within the first 12 months of corrected age.

| Weight |  |  |  | Length |  |  |  | BMD |  |  |  |
| --- | --- | --- | --- | --- | --- | --- | --- | --- | --- | --- | --- |
| Gestational week, birthweight | Infant age (month) | Mean  (kg) | SD  (kg) | Gestational week, birthweight | Infant age  (month) | Mean  (cm) | SD  (cm) | Gestational week, birthweight | Infant age (month) | Mean | SD |
| Before full term |  |  |  | Before full term |  |  |  |  |  |  |  |
| ≤ 30.9, < 1.5kg | 37.0 | 1.2 | 0.2 | ≤ 30.9, < 1.5kg | 37.0 | 43.7 | 1.1 |  |  |  |  |
| ≤ 30.9, 1.5-2.5kg | 37.0 | 1.7 | 0.2 | ≤ 30.9, 1.5-2.5kg | 37.0 | 46.0 | 0.6 |  |  |  |  |
| ≤ 30.9, < 1.5kg | 37.5 | 1.3 | 0.1 | ≤ 30.9, < 1.5kg | 37.5 | 44.5 | 0.7 |  |  |  |  |
| ≤ 30.9, 1.5-2.5kg | 37.5 | 1.8 | 0.2 | ≤ 30.9, 1.5-2.5kg | 37.5 | 47.1 | 1.1 |  |  |  |  |
| 31-36.9, 1.5-2.5kg | 38.1 | 2.2 | 0.1 | 31-36.9, 1.5-2.5kg | 38.1 | 47.6 | 1.3 |  |  |  |  |
| 31-36.9, ≥2.5kg | 38.1 | 2.5 | 0.1 | 31-36.9, ≥2.5kg | 38.1 | 50.8 | 0.3 |  |  |  |  |
| ≤ 30.9, < 1.5kg | 38.2 | 1.9 | 0.3 | ≤ 30.9, < 1.5kg | 38.2 | 45.7 | 1.6 |  |  |  |  |
| ≤ 30.9, 1.5-2.5kg | 38.2 | 2.4 | 0.3 | ≤ 30.9, 1.5-2.5kg | 38.2 | 48.7 | 0.7 |  |  |  |  |
| ≤ 30.9, < 1.5kg | 38.7 | 2.3 | 0.2 | ≤ 30.9, < 1.5kg | 38.7 | 45.7 | 0.9 |  |  |  |  |
| ≤ 30.9, 1.5-2.5kg | 38.7 | 2.3 | 0.2 | ≤ 30.9, 1.5-2.5kg | 38.7 | 48.2 | 1.3 |  |  |  |  |
| 31-36.9, 1.5-2.5kg | 38.9 | 2.3 | 0.2 | 31-36.9, 1.5-2.5kg | 38.9 | 46.8 | 0.5 |  |  |  |  |
| 31-36.9, ≥2.5kg | 38.9 | 2.9 | 0.3 | 31-36.9, ≥2.5kg | 38.9 | 48.7 | 0.8 |  |  |  |  |
| 31-36.9, 1.5-2.5kg | 39.3 | 2.8 | 0.1 | 31-36.9, 1.5-2.5kg | 39.3 | 49.0 | 1.7 |  |  |  |  |
| 31-36.9, ≥2.5kg | 39.3 | 3.1 | 0.1 | 31-36.9, ≥2.5kg | 39.3 | 52.7 | 0.8 |  |  |  |  |
| 31-36.9, 1.5-2.5kg | 39.5 | 2.8 | 0.2 | 31-36.9, 1.5-2.5kg | 39.5 | 47.9 | 0.4 |  |  |  |  |
| 31-36.9, ≥2.5kg | 39.5 | 3.5 | 0.3 | 31-36.9, ≥2.5kg | 39.5 | 49.9 | 1.0 |  |  |  |  |
| ≥ 37 , ≥2.5kg | 39.75 | 3.2 | 0.3 | ≥ 37 , ≥2.5kg | 39.75 | 50.2 | 0.2 |  |  |  |  |
| After full term |  |  |  | After full term |  |  |  | After full term |  |  |  |
| ≤ 30.9, < 1.5kg | 1 month | 3.1 | 0.3 | ≤ 30.9, < 1.5kg | 1 month | 47.6 | 1.7 | ≤ 30.9, < 1.5kg | 3 month | 0.3 | 0.1 |
| ≤ 30.9, 1.5-2.5kg | 1 month | 3.4 | 0.2 | ≤ 30.9, 1.5-2.5kg | 1 month | 51.0 | 1.5 | ≤ 30.9, 1.5-2.5kg | 3 month | 0.3 | 0.1 |
| 31-36.9, 1.5-2.5kg | 1 month | 3.6 | 0.3 | 31-36.9, 1.5-2.5kg | 1 month | 50.8 | 1.9 | 31-36.9, 1.5-2.5kg | 3 month | 0.4 | 0.1 |
| 31-36.9, ≥2.5kg | 1 month | 4.2 | 0.3 | 31-36.9, ≥2.5kg | 1 month | 52.8 | 1.2 | 31-36.9, ≥2.5kg | 3 month | 0.4 | 0.1 |
| ≥ 37 , ≥2.5kg | 1 month | 4.5 | 0.3 | ≥ 37 , ≥2.5kg | 1 month | 54.8 | 0.8 | ≥ 37 , ≥2.5kg | 3 month | 0.4 | 0.0 |
| ≤ 30.9, < 1.5kg | 2 month | 4.0 | 0.6 | ≤ 30.9, < 1.5kg | 2 month | 48.8 | 1.6 | ≤ 30.9, < 1.5kg | 6 month | 0.3 | 0.1 |
| ≤ 30.9, 1.5-2.5kg | 2 month | 4.5 | 0.5 | ≤ 30.9, 1.5-2.5kg | 2 month | 53.3 | 1.9 | ≤ 30.9, 1.5-2.5kg | 6 month | 0.3 | 0.1 |
| 31-36.9, 1.5-2.5kg | 2 month | 4.7 | 0.3 | 31-36.9, 1.5-2.5kg | 2 month | 54.0 | 1.8 | 31-36.9, 1.5-2.5kg | 6 month | 0.4 | 0.1 |
| 31-36.9, ≥2.5kg | 2 month | 5.6 | 0.5 | 31-36.9, ≥2.5kg | 2 month | 57.2 | 1.5 | 31-36.9, ≥2.5kg | 6 month | 0.4 | 0.0 |
| ≥ 37 , ≥2.5kg | 2 month | 5.7 | 0.3 | ≥ 37 , ≥2.5kg | 2 month | 58.7 | 0.8 | ≥ 37 , ≥2.5kg | 6 month | 0.5 | 0.0 |
| ≤ 30.9, < 1.5kg | 3 month | 5.1 | 0.5 | ≤ 30.9, < 1.5kg | 3 month | 52.1 | 1.9 | ≤ 30.9, < 1.5kg | 9 month | 0.4 | 0.1 |
| ≤ 30.9, 1.5-2.5kg | 3 month | 5.6 | 0.5 | ≤ 30.9, 1.5-2.5kg | 3 month | 57.0 | 1.8 | ≤ 30.9, 1.5-2.5kg | 9 month | 0.4 | 0.1 |
| 31-36.9, 1.5-2.5kg | 3 month | 5.6 | 0.6 | 31-36.9, 1.5-2.5kg | 3 month | 58.3 | 1.8 | 31-36.9, 1.5-2.5kg | 9 month | 0.4 | 0.1 |
| 31-36.9, ≥2.5kg | 3 month | 6.6 | 0.7 | 31-36.9, ≥2.5kg | 3 month | 61.3 | 1.5 | 31-36.9, ≥2.5kg | 9 month | 0.5 | 0.1 |
| ≥ 37 , ≥2.5kg | 3 month | 6.7 | 0.3 | ≥ 37 , ≥2.5kg | 3 month | 62.0 | 0.8 | ≥ 37 , ≥2.5kg | 9 month | 0.5 | 0.0 |
| ≤ 30.9, < 1.5kg | 4 month | 6.1 | 0.5 | ≤ 30.9, < 1.5kg | 4 month | 55.2 | 2.1 | ≤ 30.9, < 1.5kg | 12 month | 0.5 | 0.1 |
| ≤ 30.9, 1.5-2.5kg | 4 month | 6.5 | 0.6 | ≤ 30.9, 1.5-2.5kg | 4 month | 59.7 | 1.6 | ≤ 30.9, 1.5-2.5kg | 12 month | 0.5 | 0.1 |
| 31-36.9, 1.5-2.5kg | 4 month | 6.6 | 0.6 | 31-36.9, 1.5-2.5kg | 4 month | 61.0 | 2.0 | 31-36.9, 1.5-2.5kg | 12 month | 0.5 | 0.1 |
| 31-36.9, ≥2.5kg | 4 month | 7.4 | 0.5 | 31-36.9, ≥2.5kg | 4 month | 64.5 | 1.7 | 31-36.9, ≥2.5kg | 12 month | 0.5 | 0.0 |
| ≥ 37 , ≥2.5kg | 4 month | 7.5 | 0.3 | ≥ 37 , ≥2.5kg | 4 month | 64.7 | 0.8 | ≥ 37 , ≥2.5kg | 12 month | 0.5 | 0.0 |
| ≤ 30.9, < 1.5kg | 5 month | 6.8 | 0.5 | ≤ 30.9, < 1.5kg | 5 month | 57.1 | 1.8 |  |  |  |  |
| ≤ 30.9, 1.5-2.5kg | 5 month | 7.3 | 0.4 | ≤ 30.9, 1.5-2.5kg | 5 month | 61.6 | 1.6 |  |  |  |  |
| 31-36.9, 1.5-2.5kg | 5 month | 7.3 | 0.6 | 31-36.9, 1.5-2.5kg | 5 month | 62.8 | 1.6 |  |  |  |  |
| 31-36.9, ≥2.5kg | 5 month | 8.0 | 0.4 | 31-36.9, ≥2.5kg | 5 month | 65.9 | 1.4 |  |  |  |  |
| ≥ 37 , ≥2.5kg | 5 month | 8.1 | 0.2 | ≥ 37 , ≥2.5kg | 5 month | 66.8 | 0.6 |  |  |  |  |
| ≤ 30.9, < 1.5kg | 6 month | 7.2 | 0.4 | ≤ 30.9, < 1.5kg | 6 month | 59.9 | 1.9 |  |  |  |  |
| ≤ 30.9, 1.5-2.5kg | 6 month | 7.6 | 0.3 | ≤ 30.9, 1.5-2.5kg | 6 month | 64.5 | 1.6 |  |  |  |  |
| 31-36.9, 1.5-2.5kg | 6 month | 7.8 | 0.7 | 31-36.9, 1.5-2.5kg | 6 month | 65.0 | 1.6 |  |  |  |  |
| 31-36.9, ≥2.5kg | 6 month | 8.6 | 0.6 | 31-36.9, ≥2.5kg | 6 month | 67.8 | 1.4 |  |  |  |  |
| ≥ 37 , ≥2.5kg | 6 month | 8.5 | 0.2 | ≥ 37 , ≥2.5kg | 6 month | 68.5 | 0.5 |  |  |  |  |
| ≤ 30.9, < 1.5kg | 7 month | 7.6 | 0.4 | ≤ 30.9, < 1.5kg | 7 month | 62.2 | 1.3 |  |  |  |  |
| ≤ 30.9, 1.5-2.5kg | 7 month | 8.0 | 0.4 | ≤ 30.9, 1.5-2.5kg | 7 month | 65.6 | 1.4 |  |  |  |  |
| 31-36.9, 1.5-2.5kg | 7 month | 8.4 | 0.7 | 31-36.9, 1.5-2.5kg | 7 month | 67.0 | 1.6 |  |  |  |  |
| 31-36.9, ≥2.5kg | 7 month | 9.1 | 0.5 | 31-36.9, ≥2.5kg | 7 month | 70.1 | 1.5 |  |  |  |  |
| ≥ 37 , ≥2.5kg | 7 month | 8.8 | 0.2 | ≥ 37 , ≥2.5kg | 7 month | 70.0 | 0.3 |  |  |  |  |
| ≤ 30.9, < 1.5kg | 8 month | 7.9 | 0.3 | ≤ 30.9, < 1.5kg | 8 month | 64.1 | 1.1 |  |  |  |  |
| ≤ 30.9, 1.5-2.5kg | 8 month | 8.3 | 0.2 | ≤ 30.9, 1.5-2.5kg | 8 month | 67.4 | 1.4 |  |  |  |  |
| 31-36.9, 1.5-2.5kg | 8 month | 8.7 | 0.6 | 31-36.9, 1.5-2.5kg | 8 month | 68.4 | 1.6 |  |  |  |  |
| 31-36.9, ≥2.5kg | 8 month | 9.3 | 0.4 | 31-36.9, ≥2.5kg | 8 month | 71.1 | 1.6 |  |  |  |  |
| ≥ 37 , ≥2.5kg | 8 month | 9.2 | 0.2 | ≥ 37 , ≥2.5kg | 8 month | 71.4 | 0.3 |  |  |  |  |
| ≤ 30.9, < 1.5kg | 9 month | 8.2 | 0.4 | ≤ 30.9, < 1.5kg | 9 month | 66.4 | 1.2 |  |  |  |  |
| ≤ 30.9, 1.5-2.5kg | 9 month | 8.6 | 0.3 | ≤ 30.9, 1.5-2.5kg | 9 month | 69.4 | 1.4 |  |  |  |  |
| 31-36.9, 1.5-2.5kg | 9 month | 9.0 | 0.5 | 31-36.9, 1.5-2.5kg | 9 month | 70.3 | 1.7 |  |  |  |  |
| 31-36.9, ≥2.5kg | 9 month | 9.5 | 0.3 | 31-36.9, ≥2.5kg | 9 month | 72.4 | 1.4 |  |  |  |  |
| ≥ 37 , ≥2.5kg | 9 month | 9.5 | 0.1 | ≥ 37 , ≥2.5kg | 9 month | 72.6 | 0.4 |  |  |  |  |
| ≤ 30.9, < 1.5kg | 10 month | 8.5 | 0.3 | ≤ 30.9, < 1.5kg | 10 month | 67.5 | 1.1 |  |  |  |  |
| ≤ 30.9, 1.5-2.5kg | 10 month | 8.8 | 0.3 | ≤ 30.9, 1.5-2.5kg | 10 month | 70.3 | 1.4 |  |  |  |  |
| 31-36.9, 1.5-2.5kg | 10 month | 9.4 | 0.4 | 31-36.9, 1.5-2.5kg | 10 month | 70.8 | 1.4 |  |  |  |  |
| 31-36.9, ≥2.5kg | 10 month | 9.7 | 0.2 | 31-36.9, ≥2.5kg | 10 month | 73.5 | 1.3 |  |  |  |  |
| ≥ 37 , ≥2.5kg | 10 month | 9.8 | 0.2 | ≥ 37 , ≥2.5kg | 10 month | 73.8 | 0.3 |  |  |  |  |
| ≤ 30.9, < 1.5kg | 11 month | 8.8 | 0.3 | ≤ 30.9, < 1.5kg | 11 month | 68.5 | 1.1 |  |  |  |  |
| ≤ 30.9, 1.5-2.5kg | 11 month | 9.0 | 0.4 | ≤ 30.9, 1.5-2.5kg | 11 month | 71.1 | 1.3 |  |  |  |  |
| 31-36.9, 1.5-2.5kg | 11 month | 9.7 | 0.4 | 31-36.9, 1.5-2.5kg | 11 month | 71.9 | 1.3 |  |  |  |  |
| 31-36.9, ≥2.5kg | 11 month | 10.0 | 0.2 | 31-36.9, ≥2.5kg | 11 month | 74.4 | 1.3 |  |  |  |  |
| ≥ 37 , ≥2.5kg | 11 month | 10.0 | 0.2 | ≥ 37 , ≥2.5kg | 11 month | 75.0 | 0.3 |  |  |  |  |
| ≤ 30.9, < 1.5kg | 12 month | 9.0 | 0.3 | ≤ 30.9, < 1.5kg | 12 month | 69.6 | 1.2 |  |  |  |  |
| ≤ 30.9, 1.5-2.5kg | 12 month | 9.3 | 0.3 | ≤ 30.9, 1.5-2.5kg | 12 month | 72.2 | 1.3 |  |  |  |  |
| 31-36.9, 1.5-2.5kg | 12 month | 9.8 | 0.5 | 31-36.9, 1.5-2.5kg | 12 month | 73.5 | 1.1 |  |  |  |  |
| 31-36.9, ≥2.5kg | 12 month | 10.2 | 0.3 | 31-36.9, ≥2.5kg | 12 month | 75.4 | 1.1 |  |  |  |  |
| ≥ 37 , ≥2.5kg | 12 month | 10.3 | 0.2 | ≥ 37 , ≥2.5kg | 12 month | 76.0 | 0.4 |  |  |  |  |

Table S4. Weight, length, and bone mineral density (BMD) of adequate (AGA) or small for gestational age (SGA) preterm infants within 1 to 12 months of corrected age.

| Weight |  |  |  | Length |  |  |  | BMD |  |  |  |
| --- | --- | --- | --- | --- | --- | --- | --- | --- | --- | --- | --- |
| Infant age  (month) | Infant category | Mean  (kg) | SD  (kg) | Infant age  (month) | Infant category | Mean  (cm) | SD  (cm) | Infant age  (month) | Infant category | Mean | SD |
| Before full term | |  |  | Before full term | |  |  |  |  |  |  |
| 37.0 | SGA | 0.8 | 0.1 | 37.0 | SGA | 41.6 | 0.5 |  |  |  |  |
| 37.0 | AGA | 1.4 | 0.3 | 37.0 | AGA | 44.8 | 1.2 |  |  |  |  |
| 37.5 | SGA | 1.2 | 0.1 | 37.5 | SGA | 43.8 | 0.7 |  |  |  |  |
| 37.5 | AGA | 1.7 | 0.2 | 37.5 | AGA | 46.7 | 1.3 |  |  |  |  |
| 38.1 | SGA | 2.0 | 0.0 | 38.1 | SGA | 45.2 | 0.5 |  |  |  |  |
| 38.1 | AGA | 2.3 | 0.1 | 38.1 | AGA | 47.9 | 1.2 |  |  |  |  |
| 38.2 | SGA | 1.5 | 0.2 | 38.2 | SGA | 42.8 | 0.8 |  |  |  |  |
| 38.2 | AGA | 2.1 | 0.3 | 38.2 | AGA | 47.1 | 1.6 |  |  |  |  |
| 38.7 | SGA | 2.3 | 0.2 | 38.7 | SGA | 44.9 | 0.9 |  |  |  |  |
| 38.7 | AGA | 2.3 | 0.2 | 38.7 | AGA | 47.9 | 1.4 |  |  |  |  |
| 38.9 | SGA | 2.0 | 0.2 | 38.9 | SGA | 46.0 | 0.6 |  |  |  |  |
| 38.9 | AGA | 2.8 | 0.3 | 38.9 | AGA | 48.4 | 1.0 |  |  |  |  |
| 39.3 | SGA | 2.6 | 0.0 | 39.3 | SGA | 45.9 | 0.7 |  |  |  |  |
| 39.3 | AGA | 2.8 | 0.1 | 39.3 | AGA | 49.5 | 1.5 |  |  |  |  |
| 39.5 | SGA | 2.5 | 0.1 | 39.5 | SGA | 47.4 | 0.6 |  |  |  |  |
| 39.5 | AGA | 3.3 | 0.4 | 39.5 | AGA | 49.5 | 1.2 |  |  |  |  |
| 39.8 | SGA | 2.6 | 0.1 | 39.8 | SGA | 50.3 | 0.2 |  |  |  |  |
| 39.8 | AGA | 3.3 | 0.3 | 39.8 | AGA | 50.2 | 0.2 |  |  |  |  |
| After full term | |  |  | After full term | |  |  | After full term | |  |  |
| 1 month | SGA | 3.8 | 0.7 | 1 month | SGA | 51.1 | 4.0 | 3 month | SGA | 0.4 | 0.1 |
| 1 month | AGA | 4.1 | 0.6 | 1 month | AGA | 53.0 | 2.3 | 3 month | AGA | 0.4 | 0.1 |
| 2 month | SGA | 4.8 | 0.9 | 2 month | SGA | 54.1 | 4.6 | 6 month | SGA | 0.4 | 0.1 |
| 2 month | AGA | 5.3 | 0.6 | 2 month | AGA | 56.6 | 2.8 | 6 month | AGA | 0.4 | 0.1 |
| 3 month | SGA | 5.7 | 1.0 | 3 month | SGA | 58.1 | 4.4 | 9 month | SGA | 0.5 | 0.1 |
| 3 month | AGA | 6.3 | 0.7 | 3 month | AGA | 60.3 | 2.7 | 9 month | AGA | 0.5 | 0.1 |
| 4 month | SGA | 6.6 | 0.9 | 4 month | SGA | 60.8 | 4.3 | 12 month | SGA | 0.5 | 0.1 |
| 4 month | AGA | 7.2 | 0.6 | 4 month | AGA | 63.1 | 2.8 | 12 month | AGA | 0.5 | 0.1 |
| 5 month | SGA | 7.3 | 0.8 | 5 month | SGA | 62.9 | 4.2 |  |  |  |  |
| 5 month | AGA | 7.8 | 0.6 | 5 month | AGA | 65.0 | 2.7 |  |  |  |  |
| 6 month | SGA | 7.7 | 0.8 | 6 month | SGA | 65.0 | 3.8 |  |  |  |  |
| 6 month | AGA | 8.2 | 0.6 | 6 month | AGA | 67.0 | 2.4 |  |  |  |  |
| 7 month | SGA | 8.1 | 0.8 | 7 month | SGA | 66.7 | 3.5 |  |  |  |  |
| 7 month | AGA | 8.7 | 0.6 | 7 month | AGA | 68.7 | 2.3 |  |  |  |  |
| 8 month | SGA | 8.6 | 0.7 | 8 month | SGA | 68.1 | 3.4 |  |  |  |  |
| 8 month | AGA | 9.0 | 0.5 | 8 month | AGA | 70.1 | 2.1 |  |  |  |  |
| 9 month | SGA | 9.0 | 0.6 | 9 month | SGA | 69.7 | 3.1 |  |  |  |  |
| 9 month | AGA | 9.3 | 0.5 | 9 month | AGA | 71.6 | 1.8 |  |  |  |  |
| 10 month | SGA | 9.3 | 0.5 | 10 month | SGA | 70.8 | 3.0 |  |  |  |  |
| 10 month | AGA | 9.5 | 0.4 | 10 month | AGA | 72.6 | 1.9 |  |  |  |  |
| 11 month | SGA | 9.5 | 0.6 | 11 month | SGA | 71.9 | 3.1 |  |  |  |  |
| 11 month | AGA | 9.8 | 0.5 | 11 month | AGA | 73.7 | 2.0 |  |  |  |  |
| 12 month | SGA | 9.7 | 0.6 | 12 month | SGA | 73.3 | 2.9 |  |  |  |  |
| 12 month | AGA | 10.0 | 0.5 | 12 month | AGA | 74.8 | 1.8 |  |  |  |  |

Table S5. Differences of growth rates of weight, length, and bone mineral density (BMD) of preterm newborns when compared with full-term peers within 1 to 12 months of corrected age.

|  | Gestational age | | | | |  |
| --- | --- | --- | --- | --- | --- | --- |
|  | ≥ 37 weeks | 34-36.9 weeks | 32-33.9 weeks | 29-31.9 weeks | ≤ 28.9 weeks | *P for trend* |
| Number of participants | 1434 | 652 | 486 | 291 | 149 |  |
| *Length (cm)* |  |  |  |  |  |  |
| 1-12 months | Reference | 0.13 (0.12, 0.14) | 0.13 (0.12, 0.14) | 0.011 (0, 0.022) | 0.06 (0.046, 0.074) | < 0.001 |
| *Weight (kg)* |  |  |  |  |  |  |
| 1-12 months | Reference | 0.023 (0.022, 0.024) | 0.054 (0.053, 0.055) | 0.015 (0.014, 0.016) | 0.021 (0.019, 0.023) | < 0.001 |
| *Bone mineral density (g/cm^2^)* |  |  |  |  |  |  |
| 1-12 months | Reference | 0.0037  (0.0036, 0.0038) | 0.0057  (0.0056, 0.0058) | 0.0079  (0.0077, 0.0081) | 0.0052  (0.0050, 0.0054) | < 0.001 |

Random-effects models adjusted for mothers’ education (less than college, college or more), calcium supplementation during pregnancy (yes, no), outdoor activity during pregnancy (hour/day, continuous), gestational weight gain (kg, continuous), maternal age at birth (year, continuous), gender of the infant (male, female), birth weight (kg, continuous), and birth length (cm, continuous).

Table S6. Differences of growth rate of weight, length, and bone mineral density (BMD) of preterm newborns categorized by gestational age (GA) and birth weight (BW) within 1 to 12 months of corrected age.

|  | Gestational age and birth weight | | | | |
| --- | --- | --- | --- | --- | --- |
|  | GA: ≥ 37 weeks, BW: ≥ 2.5kg | GA: 31-36.9 weeks,  BW: ≥ 2.5kg | GA: 31-36.9 weeks,  BW: 1.5-2.5 kg | GA: ≤ 30.9 weeks,  BW: 1.5-2.5 kg | GA: ≤ 30.9 weeks,  BW: < 1.5 kg |
| Number of participants | 1414 | 474 | 661 | 266 | 174 |
| *Length (cm)* |  |  |  |  |  |
| 1-12 months | Reference | -0.63 (-0.85, -0.41) | -3.29 (-3.49, -3.09) | -4.16 (-4.45, 3.87) | -7.80 (-8.15, -7.45) |
| *Weight (kg)* |  |  |  |  |  |
| 1-12 months | Reference | -0.015 (-0.072, 0.042) | -0.65 (-0.70, -0.60) | -0.96 (-1.04, -0.88) | -1.34 (-1.44, -1.24) |
| *BMD (g/cm^2^)* |  |  |  |  |  |
| 1-12 months | Reference | -0.011 (-0.018, -0.0038) | -0.021 (-0.031, -0.011) | -0.051 (-0.065, -0.037) | -0.047 (-0.069, -0.025) |

Random-effects models adjusted for mothers’ education (less than college, college or more), calcium supplementation during pregnancy (yes, no), outdoor activity during pregnancy (hour/day, continuous), gestational weight gain (kg, continuous), maternal age at birth (year, continuous), gender of the infant (male, female), birth weight (kg, continuous), and birth length (cm, continuous).

Table S7. Differences in growth rate of weight, length, and bone mineral density (BMD) between adequate (AGA) and small for gestational age (SGA) preterm infants within 1 to 12 months of corrected age.

|  | SGA | AGA |
| --- | --- | --- |
| Number of participants | 291 | 2727 |
| *Length (cm)* |  |  |
| 1-12 months | Reference | 1.70 (1.45, 1.95) |
| *Weight (kg)* |  |  |
| 1-12 months | Reference | 0.37 (0.31, 0.44) |
| *BMD (g/cm^2^)* |  |  |
| 1-12 months | Reference | -0.010 (-0.017, -0.002) |

Random-effects models adjusted for mothers’ education (less than college, college or more), calcium supplementation during pregnancy (yes, no), outdoor activity during pregnancy (hour/day, continuous), gestational weight gain (kg, continuous), maternal age at birth (year, continuous), gender of the infant (male, female), birth weight (kg, continuous), and birth length (cm, continuous).
